# An Anodyne Formula

**Published:** 1867-09

**Authors:** 


### *An Anodyne Formula.*

The following formula is recommended for combining chloroform and morphia for internal administration: One part, by weight, of morphia is dissolved in two parts of rectified wine-vinegar, and twenty parts of rectified spirit of wine; and the solution, when cold, is mixed with eighty parts of chloroform. One drop contains the three-hundredth part of a grain of morphia. The dose for a child is two to fifteen drops; for an adult, thirty to forty drops. It is said to give relief in most painful affections much more quickly and certainly than morphia alone, and to leave none of the unpleasant after-effects of opium. The subcutaneous injection of morphia during chloroform narcosis is strongly advocated in all those cases where it is desirable to maintain the state of unconsciousness for a lengthened period.—*Med. Rec.*

